# Classification of asthma according to revised 2006 GINA: Evolution from severity to control

**DOI:** 10.4103/1817-1737.32228

**Published:** 2007

**Authors:** Emad A. Koshak

**Affiliations:** *Allergy and Clinical Immunology, Department of Internal Medicine - Faculty of Medicine - King Abdulaziz University, General Secretary of the Saudi Society of Internal Medicine, Saudi Arabia*

Asthma has grown to be one of the leading chronic illnesses throughout the world, affecting more than 300 million people of all ages. When uncontrolled, asthma can place rigorous restrictions on everyday life. On the other hand, there are so many options of therapeutic modalities including both old and more advanced medications for the control of asthma symptoms.

In order to effectively combat any chronic illness, one needs to develop and follow strategic guidelines that optimize its diagnosis, management and control. These guidelines are continuously revised and updazted according to the opinions of experts, knowledge of current scientific research, the appearance of more effective drugs or the inappropriateness in the application of a particular guideline on patients in real life.

In 1993, the Global Initiative for Asthma (GINA) was formed. Its goals and objectives were described in a 1995 NHLBI/WHO Workshop Report, Global Strategy for Asthma Management and Prevention (GSAMP). This report was revised in 2002 and its companion documents have been widely distributed and translated into many languages.

As the GINA committees expanded their work, the report was updated annually. The first update was posted in October 2003, a second in October 2004 and a third in October 2005, each including the impact of publications from January through December of the previous year.

All previous GINA reports recommended the categorization of asthmatics according to their clinical severity into four levels: Intermittent, mild persistent [Table 1]. Accordingly, this has helped asthma care providers to stratify patients to gain a clear stepwise approach of treatment options.

**Table 1:** Classification of asthma-according to severity of clinical features

Intermittent
Symptoms < once a week
Brief exacerbations
Nocturnal symptoms not > twice a month
• FEV1 or PEF ≥ 80% predicted
• PEF or FEV1 variability < 20%
Mild persistent
Symptoms > once a week but < once a day
Exacerbations may affect activity and sleep
Nocturnal symptoms > twice a month
• FEV1 or PEF ≥ 80% predicted
• PEF or FEV1 variability < 20-30%
Moderate persistent
Symptoms daily
Exacerbations may affect activity and sleep
Nocturnal symptoms > once a week
Daily use inhaled short-acting β2-agonist
• FEV1 or PEF 60-80% predicted
• PEF or FEV1 variability > 30%
Severe persistent
Symptoms daily
Frequent exacerbations
Frequent nocturnal asthma symptoms
Limitation of physical activities
• FEV1 or PEF ≤ 60% predicted
• PEF or FEV1 variability > 30%

In January 2004, the GINA executive committee recommended that the report be revised to emphasize asthma management based on clinical control rather than classification of the patient by severity. This important paradigm shift for asthma care reflects the progress that has been made in understanding the impact of the disease and the pharmacologic care of patients.

The entire, newly revised document of November 2006 emphasizes asthma control. There is now good evidence that the clinical manifestations of asthma-symptoms, sleep disturbances, limitations of daily activity, impairment of lung function and use of rescue medications-can be controlled with appropriate treatment.

The document now recommends a classification of asthma by level of control: Controlled, partly controlled or uncontrolled [Table 2]. This reflects an understanding that asthma severity involves not only the severity of the underlying disease but also its responsiveness to treatment and that severity is not an unvarying feature of an individual patient's asthma but may change over months or years.

**Table 2:** Classification of asthma-according to asthma control

Levels of asthma control			

Characteristic	Controlled (All of the following)	Partly controlled (Any measure present in anyweek)	Uncontrolled
Daytime symptoms	None (≤ twice/week)	> twice/week	Three or more features of partly controlled asthma present in any week
Limitations of activities	None	Any	
Nocturnal symptoms / awakening	None	Any	
Need for reliever/ rescue treatment	None (≤ twice/week)	> twice/week	
Lung function* (PEF or FEV1)	Normal	< 80% predicted or personal best	
Exacerbations	None	One or more/year	One in any week

* Lung function is not a reliable test for children 5 yrs and younger

Asthma control may be defined in a variety of ways. In general, the term “control” may indicate disease prevention or even cure. However, in asthma, where neither of these are realistic options at present, it refers to control of the manifestations of the disease.

Asthma control means that patients should experience no or minimal symptoms (including at night), have no limitations on their activities (including exercise), have no (or minimal) requirement for rescue medications, have near normal lung function and experience only very infrequent exacerbations [Tables 1 and 2].

Some asthma experts are not yet comfortable with the concept of allowing daytime symptoms twice a week or with the need for reliever rescue treatment twice a week. Additionally, classification of asthma based on severity, according to previous guidelines, is now reserved and recommended only for research purposes.

The role of the healthcare professional is to establish each patient's current level of control and then adjust treatment to gain and maintain control. The new document encourages channeling asthmatics into one of five, rather than four, treatment steps based on the control level, with somewhat extra flexibility and more pharmacological options than before.

This classification based on control level is by far more practical, easier to follow, includes more clinically relevant issues and implies more appropriate decisions on the choice of treatment in a given patient with asthma. In addition, telling the patient that your asthma is controlled or not controlled is more informative than intermittent or persistent asthma, especially when such words are translated into patient's own language.

According to the recent GINA document, many attempts have been made to classify asthma according to etiology, particularly with regard to environmental sensitizing agents (allergic asthma). However, such a classification is limited by the existence of patients in whom no environmental cause can be identified (non allergic asthma). Despite this, an effort to identify allergic asthma should be part of the initial assessment to enable, in some uncontrolled patients, the use of avoidance strategies, allergen-specific immunotherapy and anti-IgE therapy.

In spite of courageous efforts to improve asthma care over the past decade, a preponderance of patients have not benefited from advances in asthma management and many lack even the rudiments of care. A challenge for the next several years is to work with asthma specialists, primary healthcare providers and public health officials in each country to design, implement and evaluate asthma care programs to meet local needs.

Although many doctors dealing with asthma have some skepticism about previous or recent GINA guidelines, nevertheless it is a step forward in winning the fight against asthma.
